# A Fatal Case of Coronary Thrombosis in a Young Male Triggered by Marijuana Use

**DOI:** 10.7759/cureus.98083

**Published:** 2025-11-29

**Authors:** Akhila Aravind, Patrick B Kyle, Charulochana Subramony

**Affiliations:** 1 Department of Pathology, University of Mississippi Medical Center, Jackson, USA

**Keywords:** cannabis, cardiac arrythmia, coronary thrombosis, marijuana use, thc

## Abstract

The use of marijuana for therapeutic and recreational purposes is rising. The effects of marijuana on the central nervous system are well documented, but its effect on the cardiovascular system is not well understood. The available evidence suggests that there is an increased risk of cardiac arrhythmia and myocardial infarction with continued use of marijuana. Case reports have documented instances of ST-elevation myocardial infarction (STEMI) in otherwise healthy individuals with a history of marijuana consumption. In this study, we report a rare autopsy study of a young man with a history of marijuana use.

We report an autopsy study of a 34-year-old man with no previous medical history except for marijuana use who had a sudden cardiac death.

A complete autopsy was performed and samples for toxicological examination were obtained. Gross examination was normal except for mild cardiomegaly. Microscopic examination revealed focal complete occlusion of the proximal left anterior descending coronary artery due to an organized thrombus. The coronary arteries proximal and distal to the occlusion area showed no atherosclerotic plaques or thrombus. Trichrome stain and elastin stain were performed. There was no underlying atherosclerosis in the other coronary arteries. No acute myocardial infarction was noted. Blood and vitreous fluid toxicology revealed elevated levels of delta-9THC (tetrahydrocannabinol) (50 mg/mL).

Only rare reports of the effect of marijuana on the cardiovascular system exist in the literature. The proposed mechanisms include arteritis, vasospasm, and platelet aggregation. Our case illustrates that organized coronary thrombosis leading to coronary artery occlusion may be the cause of myocardial ischemia and fatal cardiac arrhythmia in individuals with marijuana use. This case also highlights the importance of considering coronary artery occlusion without atherosclerosis as a cause of sudden death and emphasizes the potential role of drug use in contributing to adverse cardiovascular outcomes. Cannabis smoking is a potential predisposing factor in young healthy adults.

## Introduction

Sudden cardiac death (SCD) is defined as an unexpected death due to cardiac causes occurring within an hour of symptom onset in individuals with or without previously diagnosed heart disease. SCD remains a leading cause of mortality globally, accounting for approximately 15-20% of total mortality. In most cases, it is attributed to underlying coronary atherosclerosis. However, non-atherosclerotic coronary occlusions such as coronary artery thrombosis, coronary artery spasm, and embolism, though rare, can also result in fatal outcomes. Most of the time, these cases remain undiagnosed until postmortem examination, posing diagnostic and therapeutic challenges [1].

Non-atherosclerotic coronary thrombosis is an uncommon cause of coronary occlusion that occurs without underlying atherosclerotic plaque formation. This condition has been increasingly recognized as a potential cause of SCD, particularly in younger individuals with few or no traditional cardiovascular risk factors. Hence, identification of the underlying mechanism of death in such cases is important. Among the potential causes of this condition, cannabis use has gathered special attention due to the increase in its recreational use and its association with adverse cardiovascular events.

This case report presents a unique case of fatal non-atherosclerotic coronary artery thrombus in a 34-year-old male, resulting in SCD, and highlights the role of cannabis and its metabolites in the pathogenesis of thrombosis. This case underscores the importance of detailed gross as well as histopathological evaluation in identifying the cause of death in such fatal cases and offers insights into the mechanisms underlying cannabis-related thrombotic events.

## Case presentation

A young male was found unresponsive at home by his family member. He had no significant medical history except for obesity and occasional marijuana and alcohol use. The coroner investigated the death scene and did not find any evidence to suggest foul play. There was no evidence of trauma as well. Hence, the coroner requested an autopsy without any restrictions to determine the cause of death.

External examination of the body did not show any pathologic findings or any evidence of trauma. The thorax was opened with a Y-shaped incision. Gross examination of the heart showed mild cardiomegaly (heart weight: 500 grams; mean heart weight by BMI: 292 (+40) grams), a dilated left ventricular chamber (4.4 cm), and hepatomegaly. The remainder of the organs were normal in appearance. The coronary arteries arose normally with a left dominant pattern and followed the usual distribution with no evidence of atherosclerosis. The myocardial cut surface was dark red-brown and did not show areas of softening or fibrosis. No atherosclerosis of the ascending aorta or the abdominal aorta was noted. The liver weighed 2400 grams (normal mean + standard deviation for patient: 1650 (±150) grams). Cut surfaces of the liver did not show any fibrosis, fatty changes, or metastatic lesions. The remainder of the internal organs showed no significant findings.

**Figure 1:**
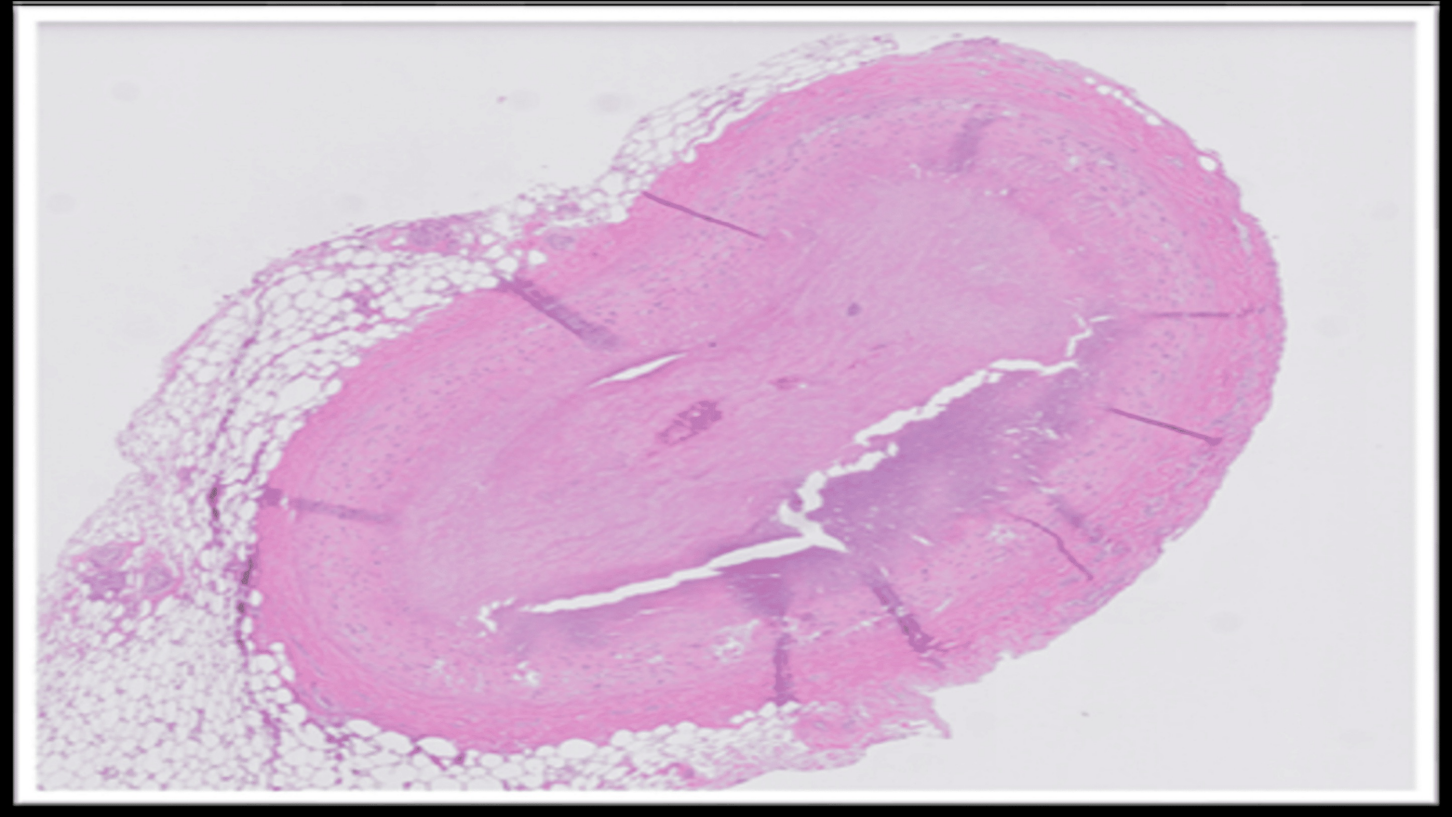
Hematoxylin and eosin section of the proximal portion of the left anterior descending coronary artery shows complete occlusion of the artery due to organized thrombus

**Figure 2:**
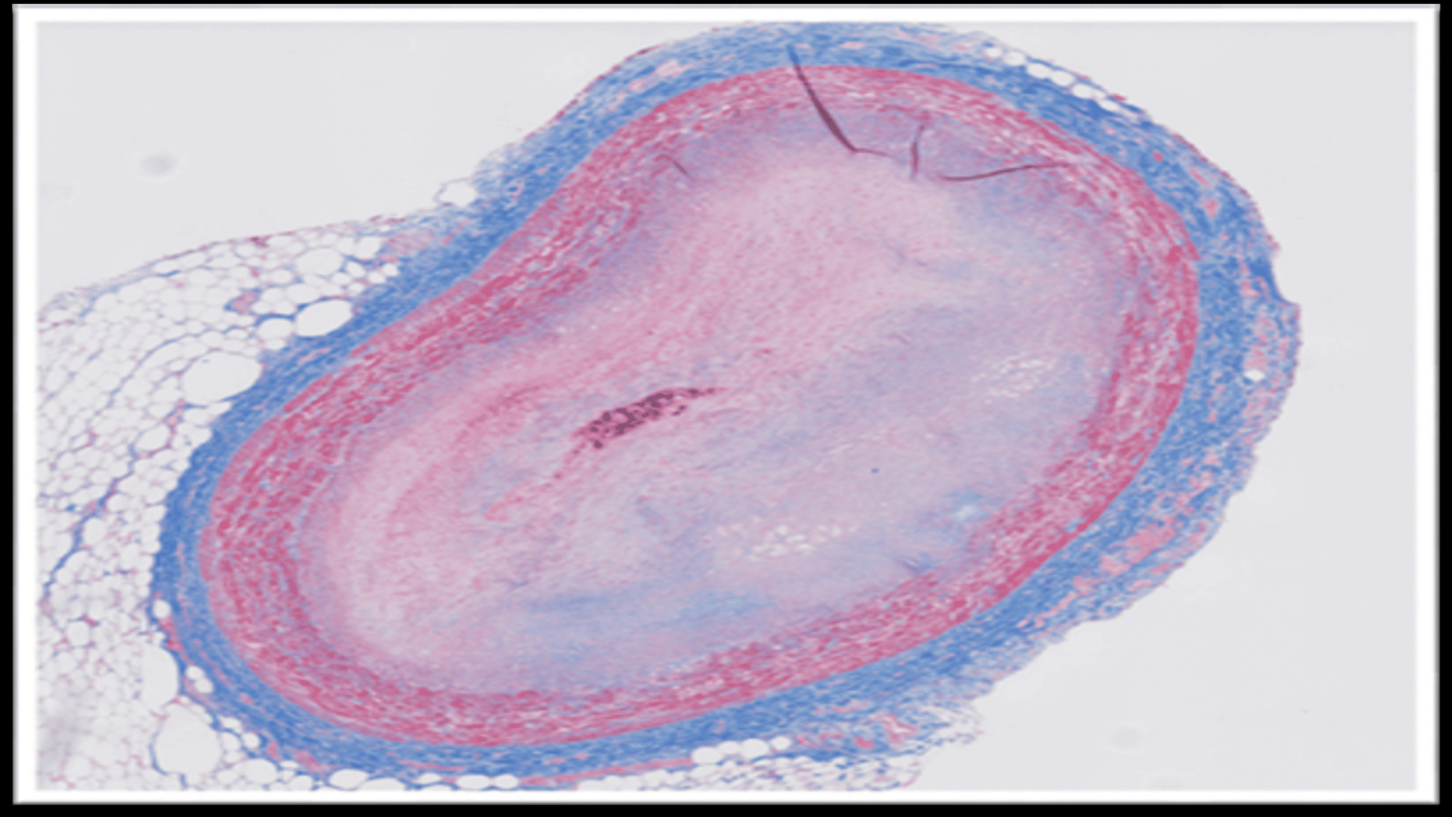
Trichrome stain shows fibroblastic growth within the thrombus

**Figure 3:**
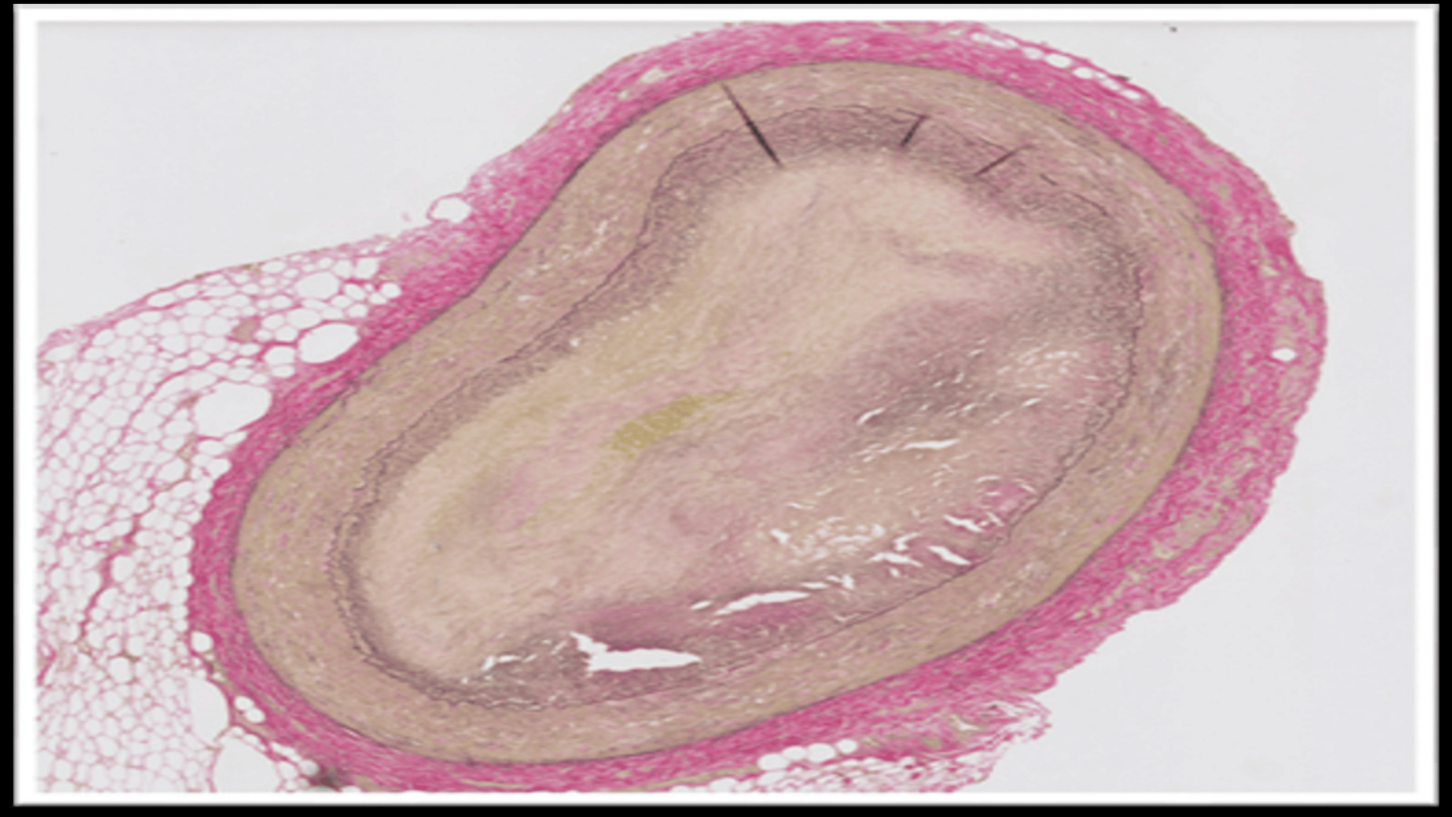
Elastin stain shows foci of disruption of the internal elastic lamina

The blood and vitreous fluid were collected for toxicology analysis. Microscopic examination of the heart showed a complete occlusion (100%) of the proximal portion of the left anterior descending coronary artery due to an organized thrombus (Figure 1). A trichrome stain was performed, which showed fibroblastic growth within the thrombus (Figure 2). Elastic stain performed on the section revealed a focus of disruption of the internal elastic lamina (Figure 3). The rest of the anterior descending coronary artery, the left circumflex artery, and the right coronary artery showed normal caliber with no evidence of thrombus or atherosclerosis. A focal area of scarring was identified in the interventricular septum and left ventricle. The right ventricular wall showed a subendocardial scar. No evidence of acute myocardial infarction (e.g., myocyte necrosis, contraction band necrosis, wavy fibers, or early neutrophilic infiltration) was identified in the sections from the right and left ventricles. The abdominal aorta showed no atherosclerotic changes. Sections from the liver showed moderate macrovesicular steatosis. No fibrosis was identified.

Microscopic examination of the rest of the organs showed no significant histopathologic alterations. Toxicology analysis of blood and vitreous by liquid chromatography-mass spectrometry (LC-MS) revealed elevated levels of metabolites of cannabis (Table 1). Although some degree of postmortem redistribution is inherent to all forensic toxicology, the use of both blood and vitreous samples, along with the markedly elevated THC metabolite concentrations, supports the reliability of these findings within the context of this autopsy case.

**Table 1:** Toxicology analysis results THC: tetrahydrocannabinol.

Drug	Patient Values	Reference Ranges
11-Hydroxy Delta-9 THC	3.3 ng/mL	1.0 ng/mL
Delta-9 carboxy THC and Delta-9 THC:	50 mg/mL	5.0 ng/mL
Delta-9 THC	28 ng/mL	0.5 ng/mL

## Discussion

Non-atherosclerotic coronary thrombosis refers to the occlusion of a coronary artery caused by a thrombus in the absence of underlying atherosclerotic plaque formation. Unlike typical coronary artery disease, which is primarily driven by atherosclerosis, this rare condition is associated with other risk factors, including drug use, vasospasm, and prothrombotic states.

Patients with non-atherosclerotic coronary thrombosis often present with SCD, as seen in the current case. Symptoms may mimic acute coronary syndrome (ACS), including chest pain, dyspnea, and arrhythmias. The absence of traditional risk factors for atherosclerosis, such as hypertension, hyperlipidemia, or diabetes, often makes the diagnosis challenging [2].

The etiology of non-atherosclerotic coronary thrombosis is multifactorial. Several mechanisms, such as vasospasm, hypercoagulable states, arteritis, and platelet aggregation, have been proposed. THC has been associated with increased cardiovascular risks, including arrhythmias, particularly in high concentrations. The available literature proposes that marijuana adversely affects the cardiovascular system mainly through three possible mechanisms: cannabis-induced arteritis, vasospasms, and platelet aggregation. However, these mechanistic pathways are derived from experimental studies or theoretical models and were not demonstrated histologically in this case; the coronary arteries, aside from the LAD thrombus, showed normal caliber and no inflammatory infiltrates.

Experimental studies have shown that cannabinoids can influence platelet function and endothelial signaling. CB1 and CB2 receptors have been identified on platelet membranes, and in vitro models suggest that high concentrations of cannabinoids such as THC may promote platelet activation and non-reversible aggregation [3,4]. The mechanism involves an indirect effect associated with the vascular wall. Once cannabinoids enter the bloodstream, parasympathetic inhibition potentially induces an inflammatory response in the arterial wall, leading to oxidative stress, endothelial erosion, and activation of factor VII events that contribute to thrombus formation [3]. The activation of CB1 receptors has also been shown to increase the expression of glycoprotein IIb-IIIa and P-selectin on platelet membranes in a dose-dependent manner, suggesting that THC can directly influence the clotting cascade, thereby initiating thrombus formation [3,4]. These findings provide a biologic framework described in prior literature but represent hypothesis-based mechanisms rather than processes demonstrated in our case.

In addition to the thrombotic findings, the patient’s obesity and cardiomegaly are important considerations, as both conditions independently increase the risk of SCD. Obesity is associated with cardiac remodeling, systemic inflammation, and a prothrombotic milieu that may predispose to arrhythmia or thrombosis even in the absence of significant atherosclerosis [5]. Cardiomegaly, likewise, has been identified in autopsy series as a frequent arrhythmogenic substrate, particularly among younger and obese individuals [6]. These factors may therefore act independently as established contributors to SCD. At the same time, a synergistic effect cannot be excluded, as structural and metabolic cardiac vulnerability may amplify the impact of any concurrent insult, such as coronary thrombosis, regardless of cannabis exposure.

In fatal cases of non-atherosclerotic coronary thrombosis, the diagnosis is established primarily through autopsy and postmortem toxicology analysis. Gross examination may not reveal significant atherosclerosis in these cases. Histopathological examination of the coronaries may reveal occlusion of the artery by thrombus. However, the possibility of a postmortem thrombus can be ruled out in such cases by doing ancillary studies like trichrome stain, which confirms fibroblast growth within the thrombus, and elastin stain, which reveals disruption of the internal elastic lamina, thereby favoring antemortem thrombus development. These histopathological findings, along with elevated levels of THC and its metabolites, can help in ruling out other causes of SCD, such as vasculitis or atherosclerosis, and thereby identifying the cause of death.

The findings in our case are consistent with the few previously reported cases of non-atherosclerotic coronary thrombi in young adults and raise the possibility of an association between chronic marijuana use, elevated THC levels, and the observed thrombus, particularly in the setting of cardiomegaly and obesity [7,8]. However, a definitive causal relationship cannot be established based on available postmortem findings.

## Conclusions

This case of focal coronary artery occlusion due to an organized thrombus underscores the importance of considering non-atherosclerotic causes of SCD, particularly in younger individuals. It also raises awareness of the potential cardiovascular effects associated with marijuana use, especially in the presence of comorbidities such as obesity and cardiomegaly. Although elevated THC levels were identified in this patient, a definitive causal relationship between marijuana exposure and thrombus formation cannot be established based on postmortem findings alone. Nevertheless, the association reported in this and other published cases highlights the need for continued investigation into the cardiovascular implications of THC, especially as its recreational use becomes increasingly common in the United States. Further studies are warranted to better understand whether and how elevated THC levels may influence thrombotic events and contribute to SCD in susceptible individuals.
